# Effect of changes in serum levels of endogenous hydrogen sulfide on fracture healing: Study protocol clinical trial (SPIRIT compliant)

**DOI:** 10.1097/MD.0000000000019684

**Published:** 2020-04-03

**Authors:** Feng Liao, Zongdong Zhu, Chengwei Xiao, Bo Tan, Xiaoming Tang, Dan Wei, Jiabin Yuan, Xuemei Xiang, Jiang Hu

**Affiliations:** a Department of Orthopaedics, Affiliated Hospital of University of Electronic Science and Technology & Sichuan Provincial People's Hospital; b Jane lab. Big Data Research Center, University of Electronic Science and Technology of China, Chengdu, China.

**Keywords:** bone turnover markers, fracture, heal, hydrogen sulfide (H_2_S)

## Abstract

**Background:**

Fracture is a common disease; many factors affect fracture healing. Recent studies have confirmed that hydrogen sulfide (H_2_S) plays an essential role in bone formation, but most of these studies are drawing conclusions based on animal experiment; whether H_2_S could promote fracture healing in patients is still unclear. We aim to investigate the change of serum H_2_S in fracture patients, and analyze its effort on fracture healing.

**Methods:**

This is a single-center, prospective cohort study. Patients with spinal or limb fracture will be recruited. Patient's serum and urine will be collected at baseline for examination (serum H_2_S, β-CTX, OC, PINP, 25-OH-VitD_3_, S-CTX, urinary calcium, and urinary creatinine). All patients will be followed-up for 24 months in outpatients settings, the image of X-ray or CT will be reviewed and fracture healing will be judged by 2 experienced orthopedic physicians. The difference in serum parameters especially H_2_S will be compared between patients with fracture healed within 9 months and those with fracture unhealed at 9 months.

**Discussion:**

Results of the trial could provide insight into influence of H_2_S on fracture healing.

**Ethics and dissemination:**

The study was approved by the ethics committee of School of Medicine UESTC & Sichuan Provincial People's Hospital Ethics Committee. All the participants will be asked to provide written informed consent before data collection. The findings of the study will be published in peer-reviewed journals and will be presented at national or international conferences.

## 1 Introduction

Fracture is common in clinical practice, healing without deformity and restoring limb function as soon as possible is the goal of fracture treatment. To date, the detailed pathophysiological mechanism of fracture healing has not been fully clarified. Previous research elucidated that the process mainly depends on the action of osteoblasts and osteoclasts, and is regulated by many cytokines, such as bone morphogenetic protein-2 (BMP-2), transforming growth factor β (TGF-β) and fibroblast growth factor (FGF).^[1,2]^

Hydrogen sulfide (H_2_S) is a gastrotransmitter and plays important regulatory roles in cardiovascular, gastrointestinal, and neurological diseases.^[3,4]^ Cystathionine b synthase (CBS), cystathionine c lyase (CSE) or 3-mercaptopyruvate sulfurtransferase are key enzymes generating H_2_S as L-cysteine as a substrate.^[5]^ In recent years, many studies have confirmed H_2_S plays an essential role in bone metabolism. H_2_S decreases osteoclast differentiation induced by nicotine or lipopolysaccharide^[6]^ and osteoclast progenitor differentiation,^[7]^ which decreases necrotic bone absorption during bone healing, but benefits osteoporosis. As well, H_2_S decreases matrix metalloproteinase activity in bone matrix,^[8]^ thereby accelerating matrix mineralization. Finally, H_2_S promotes bone marrow mesenchymal stem cells mineralization by Wnt signals.^[9]^ and reduces MC3T3-E1 preosteoblast cell injury induced by H_2_O_2._^[10]^ Our work found that CSE-H_2_S sulfhydrated Runt-related transcript factor 2 (RUNX2) enhanced its transactivation and increased osteoblast differentiation and maturation, thereby promoting rat bone healing.^[11]^ However, the above studies are based on animals or cultured cells, whether H_2_S plays similar role in patients with fracture is still unclear.

In this study, we aimed to investigate the change of serum H_2_S in fracture patients, and analyze its effort on fracture healing.

## 2 Methods and analysis

### 2.1 Study design

This is a single center, prospective, cohort study at the Sichuan Provincial People's Hospital, which is a referral hospital for trauma and has case volume necessary for the study. Trial is registered at Chinese Clinical Trial Registry (ChiCTR1900026045). This study included fracture group and control group, and is a 24-month study, which started in July 2019 and will be ended in June 2021.

**Figure 1:**
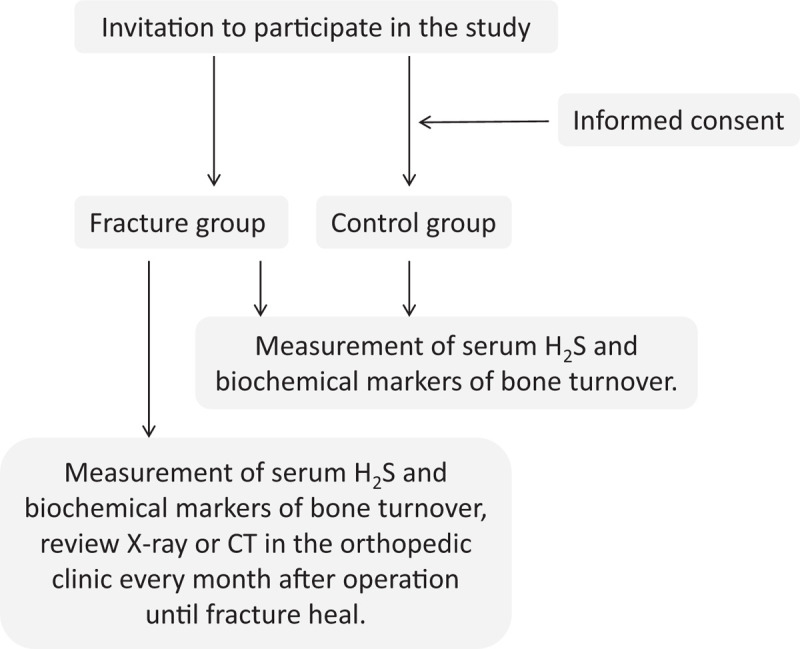
Study flowchart.

The primary outcome is concentrations of serum H_2_S. The secondary outcomes are bone turnover markers^[12,13]^ (including serum β-C-terminal telopeptide of type I collagen (β-CTX), procollagen type I N-terminal propeptide (PINP), osteocalcin (OC), serum C- terminal telopeptide of type I collagen (S-CTX),25-OH-VitD_3_ and urinary calcium/creatinine) and time between surgery and healing of the fracture. These indexes will be measured at specific time points, as described in Figure 1.

### 2.2 Participants

#### 2.2.1 Inclusion criteria

Patients with spinal or limb fracture will be recruited. Patients with fracture occurred within 3 weeks will be included, and the fracture should be confirmed by X-ray or CT. All patients (1000 patients) will be asked to provide written informed consent.

#### 2.2.2 Exclusion criteria

Patients with wound infection or serious injury requiring amputation will be excluded. Patients with a predicted survival time of less than 9 months because of other serious illness will also be excluded. Patients with pregnancy, psychological disturbance that affects outcome evaluation, or cognitive impairment will also be excluded.

### 2.3 Recruitment

Participants will be recruited from inpatient or outpatient settings in Sichuan Provincial People's Hospital. We will also recruit patients through advertise posters or local media. Patients who meet the inclusion criteria will be asked to provide written inform consent, and baseline evaluation including basic information collection, measurement of bone metabolic markers, measurement of serum H_2_S will be performed subsequently.

### 2.4 Data collection

#### 2.4.1 Basic information

Basic information such as patient age, gender, and fracture site will be extracted from the hospital medical record system. We will record the basic information of the included participants, which include age, gender, location of facture, smoking, frequency and mode of exercise, accompanied disease, long-term medication (name of medication, dose, and frequency of administration), and other conditions that related to bone fracture.

#### 2.4.2 Measurement of bone metabolic markers

Serum β-CTX, OC, PINP, 25-OH-VitD_3_, and S-CTX were measured by using Elisa method (Shanghai Zhuocai Biological Technology Co., Ltd., China). Urinary calcium and creatinine were measured with automatic biochemical analyzer (Hitachi 7600, Japan).

#### 2.4.3 Measurement of serum H_2_S

We will use modified methylene blue method for measurement of H_2_S level.^[14]^ We will add Zn^2+^ to serum sample to deposit H_2_S, HS^−^, S^2−^, and plasma protein; NaOH will then be used to re-dissolve plasma protein. ZnS deposition was re-dissolved by the addition of N, N-dimethyl-p-phenylenediamine, and the remnant protein was deposited by trichloroacetic acid. After centrifugation, ferriammonium sulfate was added to the supernatant fluid to generate methylene blue, which was analyzed by spectrophotometer at 665 nm.

### 2.5 Outcomes

Standard for fracture healing:

(1) No pain in the fracture part and no pain along the longitudinal axis.
(2) Raise the limb without discomfort.
(3) Use proper force to reverse the affected limb and no abnormal activity in the fracture.
(4) The injured limb can meet the following requirements to lift 1 kg weight to 1 minute; The lower extremity can walk 3 minutes without holding on the ground, not less than 30 steps. Continuous observation of 2 weeks of fracture without deformation.
(5) X-ray shows the fracture line blurred, with continuous callus through the fracture line.^[15,16]^ Fracture healing was defined as being up to the all above standards.

Fracture healing is judged by 2 experienced orthopedic physicians, which usually takes 4 to 8 months after reduction and fixation, and if not heal for more than 9 months, it is considered delayed or non-union.^[17]^

### 2.6 Planned statistical analysis

The analysis will be conducted by Statistical Package for the Social Sciences (SPSS) Statistics software, version 19.0 (IBM Corporation, Armonk, NY). Continuous data will be presented as the mean ± standard deviation (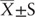), while categorical data will be presented as raw numbers and frequencies. Kolmogorov-Smirnov and Shapiro-Wilk tests will be used to test whether data distribution is normal. Data with normal distribution will be analyzed by Student *t* test. Non-parametric tests Mann–Whitney *U* test will be used to assess the difference between patients with healed fracture and those without when data distribution is skewed. Logistic regression model will be developed with whether fracture is healed at 9 months as dependent variable, with level of H_2_S as independent variable, and with age, gender, baseline characteristics, and other parameters as covariates. *P* < .05 is considered statistical significance.

## 3 Discussion and practical implications

Increasing data suggest that H_2_S plays an essential role in bone metabolism. Our previous study found that overexpressed CSE promoted bone fracture healing through increasing collagen secretion, promoting endochondral ossification, and building a full bridge between fracture sites in rat femur.^[11]^ However, whether endogenous H_2_S has the same effect on patients with fracture is still unclear. Therefore, we design an observational study to investigate the relationship between serum H_2_S concentration and bone fracture healing.

The study is an important and innovative prospective cohort study that will provide insight into influence of H_2_S on fracture healing, which, to the best of our knowledge, is the first study in human. The findings may have important implications for therapeutic strategies targeted at promoting healing of fractures.

## 4 Ethics and dissemination

The study was approved by the ethics committee of School of Medicine UESTC & Sichuan Provincial People's Hospital Ethics Committee. All the participants gave written informed consent before data collection. The findings of the study will be published in peer-reviewed journals and will be presented at national or international conferences.

## Acknowledgments

The authors are grateful for the help from all the colleagues and co-workers from the Department of Orthopedics, Affiliated Hospital of University of Electronic Science and Technology & Sichuan Provincial People's Hospital.

## Author contributions

**Data curation:** Feng Liao, Zongdong Zhu, Chengwei Xiao, Bo Tan, Xiaoming Tang, Dan Wei

**Formal analysis:** Feng Liao

**Methodology:** Feng Liao

**Project administration:** Feng Liao

**Supervision:** Jiang Hu

**Writing – original draft:** Feng Liao

**Writing – review & editing:** Feng Liao, Jiabin Yuan, Jiang Hu

Jiang Hu orcid: 0000-0002-9840-9749.
